# Real‐World Treatment Patterns and Clinical Outcomes Among Patients With Triple‐Class–Exposed and BCMA‐Exposed Multiple Myeloma Within the United States

**DOI:** 10.1002/jha2.70145

**Published:** 2025-09-23

**Authors:** Hira S. Mian, Jennifer S. Harper, Hoa H. Le, Alex Z. Fu, Saurabh Patel, Xinke Zhang, Rafael Fonseca

**Affiliations:** ^1^ Department of Oncology McMaster University Hamilton Ontario Canada; ^2^ Johnson & Johnson Titusville New Jersey USA; ^3^ Johnson & Johnson Horsham Pennsylvania USA; ^4^ Division of Hematology and Medical Oncology Mayo Clinic Phoenix Arizona USA

**Keywords:** BCMA, clinical outcomes, multiple myeloma, real‐world, triple‐class–exposed

## Abstract

**Introduction:**

A novel therapy for heavily pretreated triple‐class–exposed multiple myeloma (TCE MM) is B‐cell maturation antigen (BCMA)‐targeted immunotherapy. While the number of TCE+BCMA‐exposed patients is growing, real‐world data for this group are limited.

**Methods:**

We present real‐world data from patients with TCE+BCMA‐exposed MM who initiated a subsequent line of therapy (LOT) using a US‐based claims database, Komodo's Healthcare Map.

**Results:**

We identified 656 TCE+BCMA‐exposed patients; mean age was 66.5 years. Time from MM diagnosis to index was 5.4 years; mean number of prior LOTs was 5.9. The most prevalent prior therapy received within each drug class was daratumumab (98.5%), pomalidomide (86.0%), carfilzomib (85.8%) and belantamab mafodotin (74.5%). A total of 137 different subsequent treatment regimens were observed following TCE+BCMA exposure; the most common regimen was teclistamab (10.4%). The top three targeted agents within the subsequent regimen were carfilzomib (20.2%), pomalidomide (20.1%) and bortezomib (16.6%). Among this TCE+BCMA‐exposed population who received subsequent treatment, the median time to next treatment or death was 6.8 (95% CI, 6.1–7.5) months; time to treatment discontinuation or death was 3.5 (95% CI, 3.2–3.7) months.

**Conclusion:**

This first real‐world analysis of patients with heavily pretreated TCE+BCMA‐exposed MM shows poor clinical outcomes, frequent therapy retreatment and no standard‐of‐care, highlighting the need for novel treatments.

**Clinical Trial Registration:**

The authors have confirmed clinical trial registration is not needed for this submission.

## 1 Introduction

The introduction of proteasome inhibitors (PIs), immunomodulatory drugs (IMiDs) and monoclonal antibodies (mAbs) has resulted in markedly improved disease outcomes, including higher overall survival (OS) rates, among patients with multiple myeloma (MM) [1]. However, despite this, MM remains an incurable disease, with patients ultimately experiencing disease relapse and progression [2].

Patients with MM who experience disease relapse or become refractory to initial treatments often require subsequent lines of therapy (LOTs) for continued disease management. However, each successive LOT has been associated with reduced efficacy, duration of response and OS [3]. Patients with MM that is triple‐class–exposed (TCE; defined as exposure to ≥ 1 PI, ≥ 1 IMiD and ≥ 1 anti‐CD38 mAb) have particularly poor outcomes. Retrospective real‐world analyses using claims‐based data and electronic health record (EHR) databases estimated that median time to treatment discontinuation or death was 4.2–5.3 months [4, 5], and median time to next treatment (TTNT) ranged from 1.7–7.8 months among heavily pretreated patients with TCE MM [4, 5, 6].

Recently, several innovative therapies targeting B‐cell maturation antigen (BCMA) have emerged as promising options for heavily pretreated patients with TCE MM. BCMA is crucial for B‐cell proliferation and plasma cell differentiation and is found in higher proportions on myeloma cells than on non‐malignant plasma cells, making it a key target of interest [7]. Three major types of BCMA‐targeting therapies include antibody‐drug conjugates, bispecific antibodies and chimeric antigen receptor T‐cell (CAR‐T) therapies. The first BCMA‐targeted antibody‐drug conjugate, belantamab mafodotin (belantamab), was initially approved by the US Food and Drug Administration (FDA) based on the DREAMM‐2 trial but was later withdrawn due to unmet Phase 3 trial requirements [8, 9, 10]. Since then, several other BCMA‐targeting therapies have been approved for patients with TCE MM in the later‐line setting, including the bispecific antibodies teclistamab [11, 12] and elranatamab [13] and the CAR‐T therapies idecabtagene vicleucel (ide‐cel) [14, 15] and ciltacabtagene autoleucel (cilta‐cel) [16, 17], all of which demonstrated promising outcomes, thus leading to their FDA approval.

An increasing number of patients with TCE MM are now being exposed to BCMA‐targeting agents, resulting in a new category of patients who are both TCE‐ and BCMA‐exposed. However, despite this increasingly prevalent patient population, there are limited real‐world data on outcomes for patients who are TCE‐ and BCMA‐exposed. To understand treatment patterns and outcomes, real‐world evidence is crucial, as such analyses may provide insight into unmet needs for this patient population and the optimal subsequent treatment. Here we present a retrospective, observational, descriptive cohort study using a large, US‐based claims database to describe characteristics, treatment patterns and clinical outcomes of patients with TCE‐ and BCMA‐exposed MM who initiated a subsequent LOT.

## 2 Methods

### 2.1 Data Source

This study leveraged data, inclusive of medical (inpatient/outpatient) and pharmacy claims, gathered from Komodo's Healthcare Map dataset, which spans over 150 national payors, consortiums and nonpayor sources within the United States. Komodo's Healthcare Map includes open and closed health insurance claims data from over 330 million individuals throughout the United States, with ∼160 million individuals with both medical and pharmacy claims [18]. Closed claims within this dataset represent those that have undergone insurance adjudication and are sourced from payors that provide eligibility and enrolment information. The distribution of payor type for closed claims is ∼65% commercial, ∼7% Medicare Advantage and ∼28% managed Medicaid. Open claims include additional payor channels such as Medicare, Medicaid, Veterans Affairs and TRICARE, but do not provide enrolment information. Both open and closed claims were utilised in this analysis. To ensure patient confidentiality, Komodo Health utilises third‐party data logistics (Datavant) for deidentified linking of patient data across sources.

### 2.2 Study Design

**FIGURE 1:**
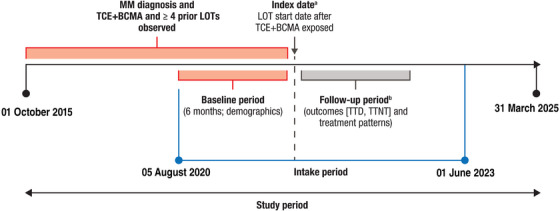
Study design for patients with TCE+BCMA‐exposed MM. BCMA, B‐cell maturation antigen; CAR‐T, chimeric antigen receptor T‐cell; LOT, line of therapy; MM, multiple myeloma; TCE, triple‐class–exposed; TTD, time to discontinuation; TTNT, time to next treatment. ^a^Index date was defined as the start date of the subsequent LOT following TCE+BCMA exposure (or CAR‐T infusion date for CAR‐T‐containing LOTs). ^b^The follow‐up period was defined as the time from the index date to the last claim date, death or the end of the study period, whichever was earlier.

This retrospective, observational, descriptive cohort study included data from 1 October 2015 to 31 March 2025. Patients were identified for inclusion during the ‘intake period’ (the allowed range for the index date): as early as 5 August 2020 (FDA approval date of belantamab, the first BCMA‐targeted therapy), and as late as 1 June 2023 (1 year before study end date, to allow sufficient follow‐up time; Figure 1). The population of interest was patients with TCE‐ and BCMA‐exposed MM who had received ≥ 4 prior LOTs and initiated a subsequent LOT. Patients with diagnosed MM, per International Classification of Diseases (ICD) diagnosis codes, were first identified from the database. Patients were required to have TCE and BCMA exposure between the study start date and intake end date. The first subsequent LOT following TCE and BCMA exposure was defined as the ‘index LOT’, and the date of initiation defined as the ‘index date’. However, if the index LOT contained a CAR‐T therapy, then the index date was the CAR‐T infusion date instead of the LOT start date (which may be the start of bridging therapy before the main CAR‐T treatment). The ‘baseline’ period was defined as the 6 months immediately preceding the index date. The ‘follow‐up’ period was defined as the time from the index date to the last claim date, death, or the end of the study period, whichever was earlier.

### 2.3 Line of Therapy Algorithm

To derive LOTs, a previously reported algorithm was utilised with some key modifications [19]. Briefly, a regimen is formed by ≥ 1 National Comprehensive Cancer Network–recommended medications for MM identified from medical and pharmacy claims during the first 60 days following the regimen initiation. A treatment gap was defined as the days elapsed between the last day of supply of a dispensing and its subsequent dispensing. Discontinuation of an individual medication was defined as the failure to refill the medication within the maximum allowed gap of 90 days (exceptions: [1] if a stem cell transplant [SCT] occurred prior to the end of a gap, another 6‐month gap was allowed after the SCT; [2] a 6‐month gap was permitted for belantamab to account for adverse event management). Discontinuation of a regimen was defined as discontinuation of all medications in the given regimen (excluding corticosteroids) or a change in treatment (switching or augmenting with a different drug from the regimen), whichever occurred first. Regimens were sequenced into LOTs (i.e., maintenance regimens did not advance LOTs). Given the emergence of CAR‐T therapy, specific considerations were made for CAR‐T LOTs (e.g., bridging therapy received before CAR‐T was combined into the same LOT as CAR‐T). Unspecified CAR‐T codes potentially denoted the administration of any CAR‐T therapy, encompassing treatments designated for diseases beyond MM. Within the context of patients with TCE MM, the assumption was that such unspecified codes, when used in claims after the FDA approval of ide‐cel, can be interpreted as indicative of treatment with either ide‐cel or cilta‐cel. Unspecified CAR‐T claims from 26 March 2021 (ide‐cel approval date) to the day before 28 February 2022 (cilta‐cel approval date) were assumed to represent treatment with ide‐cel.

### 2.4 Study Population

Eligible patients were aged ≥ 18 years at the index date, had ≥ 1 MM diagnosis code (ICD‐9‐CM code 203.0x or ICD‐10‐CM code C90.0x) any time prior to or on the index date, had TCE MM, had prior exposure to BCMA‐targeted treatment, and received ≥ 1 subsequent LOT following TCE and BCMA exposure. Patients were required to have ≥ 4 LOTs before index and have both medical and pharmacy claims for a MM drug ≥ 6 months before index. Exclusion criteria included death date before index date and clinical trial enrolment on the index date. For the main analysis, because the data source contains both open and closed claims, patients were not required to have continuous enrolment to obtain a larger, more representative cohort. Exploratory and supplemental analyses were conducted for patients without and with penta‐exposure (≥ 2 PIs, ≥ 2 IMiDs, ≥ 1 anti‐CD38 mAb) and patients who had received a BCMA‐targeted therapy in the index LOT. An additional exploratory and supplemental analysis with continuous enrolment was also conducted, with continuous enrolment defined as enrolment in both medical and pharmacy plans from 6 months before the LOT start date through the LOT start date (baseline period).

### 2.5 Outcomes

Patient demographic and clinical characteristics were measured on index date, including age, sex, race, region, duration of MM diagnosis, Quan–Charlson Comorbidity Index score and comorbidities. Treatment history and patterns were also determined. Treatment patterns were reported among patients with ≥ 3 months post‐index activity (unless death was observed before last claim date); patients omitted from the treatment pattern analysis may not have sufficient follow‐up to identify the combination regimen based on the LOT algorithm. Clinical outcomes included TTNT, defined as time from the index date to death or the initiation of a next LOT, and time to discontinuation (TTD), defined as time from the index date to death or the discontinuation of the index LOT.

### 2.6 Analysis

All study variables were examined descriptively. Means and standard deviations (SDs) were reported for continuous variables; numbers and percentages were reported for categorical variables. Time‐to‐event outcomes were assessed using Kaplan–Meier analyses. For TTNT, censoring events included clinical trial enrolment, last claim date, end of study period and SCT. For TTD, censoring events included clinical trial enrolment, last claim date, end of study period, SCT, or next LOT or death not observed and LOT end date within 90 days of the last claim date or end of study period. CAR‐T regimens were excluded from the TTD analysis (not applicable). For the exploratory analysis of TTNT and TTD in the continuous enrolment cohort, additional censoring events included continuous enrolment end date and last closed claim date. Per Komodo Health's deidentification policies, patient counts of 1–10 were masked.

## 3 Results

### 3.1 Patients

**FIGURE 2:**
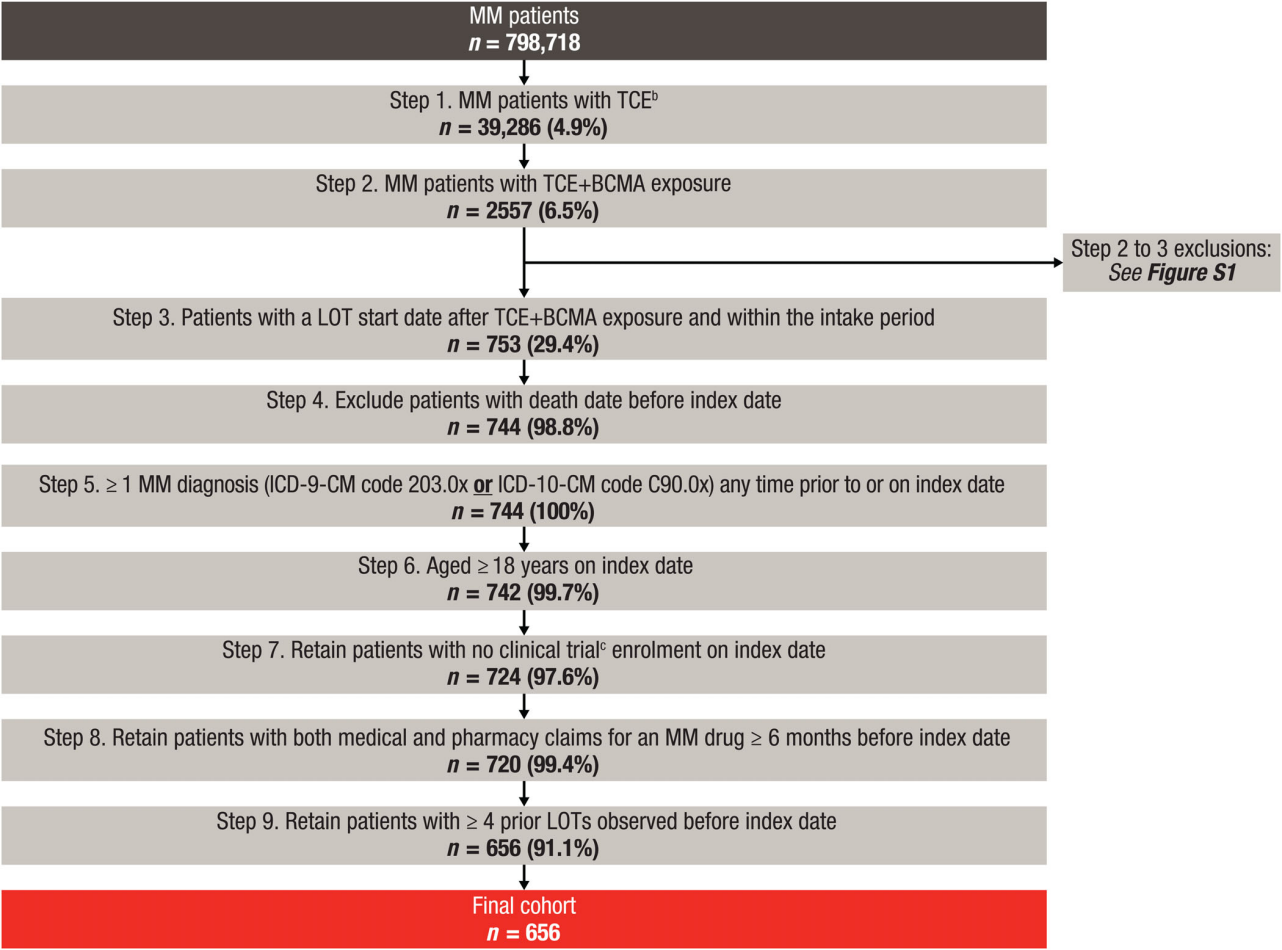
Summary of attrition and cohort selection for patients with MM with prior TCE+BCMA exposure and ≥ 4 prior LOTs. BCMA, B‐cell maturation antigen; HCPCS, Healthcare Common Procedure Coding System; ICD‐9‐CM, *International Classification of Diseases, Ninth Revision, Clinical Modification*; ICD‐10‐CM, *International Classification of Diseases, Tenth Revision, Clinical Modification*; IMiD, immunomodulatory drug; LOT, line of therapy; mAb, monoclonal antibody; MM, multiple myeloma; PI, proteasome inhibitor; TCE, triple‐class–exposed. ^a^Retention percentages are calculated from the total number of patients from the previous step. ^b^TCE was defined as ≥ 1 PI, ≥ 1 IMiD and ≥ 1 anti‐CD38 mAb between the study start date and intake end date. ^c^Codes used for clinical trials were as follows: ICD‐9‐CM: V70.7; ICD‐10‐CM: Z00.6; HCPCS: S9988, S9990, S9991, S9992, S9994, S9996; HCPCS modifier: Q0, Q1.

A total of 798,718 patients had ≥ 1 MM diagnosis code (Figure 2). Of these, 39,286 had TCE MM, of which 2557 were TCE+BCMA‐exposed (including exposure to belantamab [*n* = 1247], ide‐cel [*n* = 544], cilta‐cel [*n* = 222], unspecified CAR‐T [ide‐cel or cilta‐cel; *n* = 155] and teclistamab [*n* = 389]; Figure 2 and Figure S1). Among the 2557 patients with TCE+BCMA‐exposed MM, 753 had a subsequent LOT after TCE+BCMA exposure and within the intake period. A total of 656 patients met all subsequent criteria and were included. Mean (SD) duration of follow‐up was 17.3 (12.5) months.

Demographic and clinical characteristics for patients with TCE+BCMA‐exposed MM (*n* = 656) are presented in Table 1. Mean age at index date was 66.5 years, 57.9% (380/656) were male and 19.5% (128/656) were Black. Mean Quan–Charlson Comorbidity Index score was 3.7; the most common comorbidity was any CRAB symptom, defined as the presence of any of the following: hypercalcaemia, renal impairment, anaemia or bone lesions (60.5% [397/656]).

**TABLE 1:** Demographic and clinical characteristics and treatment history in patients with MM with prior TCE+BCMA exposure and ≥ 4 prior LOTs.

	TCE+BCMA (*n* = 656)
Mean (SD) age, years	66.5 (9.9)
Sex, *n* (%)	
Male	380 (57.9)
Female	276 (42.1)
Race, *n* (%)	
White	397 (60.5)
Black	128 (19.5)
Hispanic or Latino	50 (7.6)
Asian or Pacific Islander	19 (2.9)
Other or unknown	35 (5.3)
US region, *n* (%)	
South	232 (35.4)
Northeast	165 (25.2)
Midwest	139 (21.2)
West	85 (13.0)
Other^a^	35 (5.3)
Mean (SD) QCCI score	3.7 (3.2)
Comorbidities, *n* (%)	
Any CRAB symptom	397 (60.5)
Hypertension	395 (60.2)
Peripheral neuropathy	275 (41.9)
Cardiovascular conditions	195 (29.7)
Diabetes	146 (22.3)
Mean (SD) time from first MM diagnosis to index date, years	5.4 (1.5)
Mean (SD) prior LOTs before index date	5.9 (1.6)
Mean (SD) time from prior LOT end date to index date, months	3.9 (4.5)
Prior treatments before index date	
Prior PI, *n* (%)	
Carfilzomib	563 (85.8)
Bortezomib	510 (77.7)
Ixazomib	170 (25.9)
Prior IMiD, *n* (%)	
Pomalidomide	564 (86.0)
Lenalidomide	470 (71.6)
Thalidomide	47 (7.2)
Prior anti‐CD38 mAb, *n* (%)	
Daratumumab	646 (98.5)
Isatuximab	72 (11.0)
Prior BCMA, *n* (%)	
Belantamab	489 (74.5)
Mean (SD) duration of prior belantamab treatment, days	105 (133.8)
Ide‐cel	134 (20.4)
Unspecified CAR‐T^b^	20 (3.0)
Teclistamab	< 11 (< 1.7)^c^
Cilta‐cel	< 11 (< 1.7)^c^
Prior selinexor, *n* (%)	136 (20.7)
Prior SCT, *n* (%)	333 (50.8)
Penta‐drug–exposed,^d^ *n* (%)	316 (48.2)

Abbreviations: BCMA, B‐cell maturation antigen; belantamab, belantamab mafodotin; CAR‐T, chimeric antigen receptor T‐cell; cilta‐cel, ciltacabtagene autoleucel; CRAB, hypercalcaemia, renal failure, anaemia, bone lesions; ide‐cel, idecabtagene vicleucel; IMiD, immunomodulatory drug; LOT, line of therapy; mAb, monoclonal antibody; MM, multiple myeloma; PI, proteasome inhibitor; QCCI, Quan–Charlson Comorbidity Index; SCT, stem cell transplant; SD, standard deviation; TCE, triple‐class–exposed.

^a^ Other is defined as 2+ regions.

^b^ Ide‐cel or cilta‐cel.

^c^ Due to Komodo Health's patient deidentification policies, data including 1–10 patients were masked.

^d^ Defined as having received ≥ 2 PIs, ≥ 2 IMiDs and ≥ 1 anti‐CD38 therapy.

Mean time from first observed MM diagnosis to index date was 5.4 years (Table 1). The mean number of prior LOTs before index date was 5.9; mean time from prior LOT end date to index date was 3.9 months. The most prevalent prior PI was carfilzomib (85.8% [563/656]), IMiD was pomalidomide (86.0% [564/656]), anti‐CD38 mAb was daratumumab (98.5% [646/656]) and BCMA was belantamab (74.5% [489/656]). Half of all patients (50.8% [333/656]) had prior SCT, and almost half were penta‐exposed (48.2% [316/656]).

### 3.2 Treatment Patterns

A total of 633 patients with TCE+BCMA‐exposed MM were included in the treatment pattern analysis. A summary of index LOT treatment patterns is provided in Table 2. After excluding corticosteroids, 137 different regimens were identified; the most common regimens were teclistamab (10.4% [66/633]), pomalidomide (5.1% [32/633]), ide‐cel (4.7% [30/633]), selinexor (3.9% [25/633]) and carfilzomib plus cyclophosphamide (3.6% [23/633]). Aside from corticosteroids, the most frequent drug class within index LOT regimens was PIs (38.4% [243/633]), followed by BCMA‐targeting immunotherapies (29.4% [186/633]), IMiDs (24.5% [155/633]), chemotherapy (21.2% [134/633]), selective inhibitors of nuclear export (16.3% [103/633]) and anti‐CD38 mAbs (16.1% [102/633]; Table 2). All patients who received a PI, IMiD, anti‐CD38 mAb or BCMA‐targeting immunotherapy in the index LOT had previously been treated with that same drug class prior to the index LOT (Table S1). When looking at individual drugs within regimens, dexamethasone (66.0% [418/633]), carfilzomib (20.2% [128/633]), pomalidomide (20.1% [127/633]), bortezomib (16.6% [105/633]), selinexor (16.3% [103/633]), teclistamab (12.6% [80/633]) and cyclophosphamide (12.6% [80/633]) were the most common.

**TABLE 2:** Index LOT treatment patterns^a^ in patients with MM with prior TCE+BCMA exposure and ≥ 4 prior LOTs.

	TCE+BCMA (*n* = 633)
Most common drug classes within index regimen,^b^ %	
Corticosteroid	433 (68.4)
PI	243 (38.4)
BCMA	186 (29.4)
IMiD	155 (24.5)
Chemotherapy	134 (21.2)
SINE	103 (16.3)
Anti‐CD38 mAb	102 (16.1)
Anti‐CS1	39 (6.2)
Most common drugs within index regimen,^b^ %	
Dexamethasone	418 (66.0)
Carfilzomib	128 (20.2)
Pomalidomide	127 (20.1)
Bortezomib	105 (16.6)
Selinexor	103 (16.3)
Teclistamab	80 (12.6)
Cyclophosphamide	80 (12.6)
Daratumumab	52 (8.2)
Isatuximab	50 (7.9)
Ide‐cel	46 (7.3)
Belantamab	45 (7.1)
Prednisone	39 (6.2)
Elotuzumab	39 (6.2)
Bendamustine	34 (5.4)
Most common index regimens,^c^, ^d^ *n* (%)	
Teclistamab	66 (10.4)
Pomalidomide	32 (5.1)
Ide‐cel	30 (4.7)
Selinexor	25 (3.9)
Carfilzomib + cyclophosphamide	23 (3.6)
Bortezomib + selinexor	22 (3.5)
Carfilzomib	21 (3.3)
Belantamab	21 (3.3)
Bortezomib	18 (2.8)
Bendamustine	15 (2.4)
Carfilzomib + selinexor	15 (2.4)
Carfilzomib + isatuximab	15 (2.4)
Belantamab + pomalidomide	14 (2.2)
Isatuximab + pomalidomide	14 (2.2)
Elotuzumab	13 (2.1)
Melphalan	13 (2.1)

Abbreviations: BCMA, B‐cell maturation antigen; belantamab, belantamab mafodotin; ide‐cel, idecabtagene vicleucel; IMiD, immunomodulatory drug; LOT, line of therapy; mAb, monoclonal antibody; MM, multiple myeloma; PI, proteasome inhibitor; SINE, selective inhibitor of nuclear export; TCE, triple‐class–exposed.

^a^ Among patients with ≥ 3 months post‐index activity (unless death was observed before last claim date).

^b^ Most common is defined as a frequency of ≥ 5% of patients.

^c^ Most common is defined as a frequency of > 2% of patients.

^d^ Regimen names after excluding corticosteroids.

### 3.3 Clinical Outcomes

**FIGURE 3:**
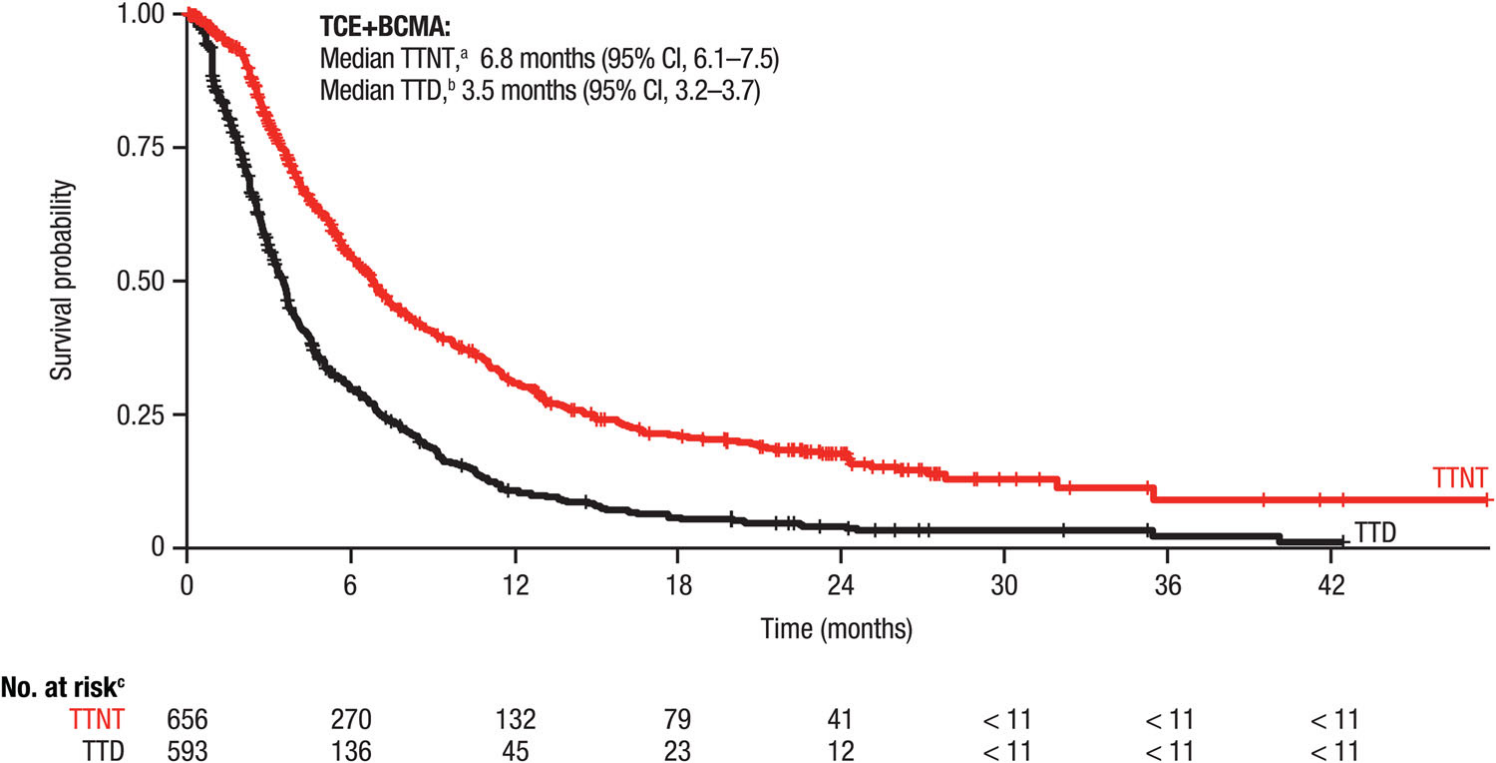
TTNT and TTD in patients with MM with prior TCE+BCMA exposure and ≥ 4 prior LOTs. BCMA, B‐cell maturation antigen; CI, confidence interval; LOT, line of therapy; MM, multiple myeloma; TCE, triple‐class–exposed; TTD, time to discontinuation; TTNT, time to next treatment. ^a^TTNT was defined as the time from the index date to death or the initiation of a next LOT. ^b^TTD was defined as the time from the index date to death or the discontinuation of the index LOT. ^c^Due to Komodo Health's patient deidentification policies, data including 1–10 patients were masked.

Among the 656 patients with TCE+BCMA‐exposed MM, 35.4% were censored from TTNT analyses. Median TTNT was 6.8 (95% CI, 6.1–7.5) months from the index date (Figure 3). Of the 191 patients with BCMA‐targeted therapy in the index LOT (Table S2), median TTNT was 11.7 (95% CI, 8.7–15.0) months (Figure S2). Among the 593 patients included in the TTD analysis, 19.6% were censored. Median TTD was 3.5 (95% CI, 3.2–3.7) months (Figure 3). Similar trends in clinical outcomes were generally observed for subgroups of patients with TCE+BCMA‐exposed MM, including those without penta‐exposure, those with penta‐exposure and those retreated with BCMA therapy in the index LOT (Figures S2–S4).

A separate, exploratory analysis was also conducted that required continuous enrolment during the baseline period. In this analysis, 194 patients were identified with TCE+BCMA‐exposed MM and continuous enrolment. Compared to the main analysis without continuous enrolment, observed TTNT and TTD were similar to that with continuous enrolment (Table S3).

Among the 656 patients with TCE+BCMA‐exposed MM, 54.3% were censored from OS analyses. Median OS was 26.9 (95% CI, 24.3–34.3) months from the index date. Data on deaths were not fully captured and, thus, this OS may be overestimated.

## 4 Discussion

In this large, real‐world study, we analysed characteristics, treatment patterns and clinical outcomes of patients with heavily pretreated MM and prior TCE and BCMA exposure who received a subsequent LOT. Data presented here demonstrate the lack of standard‐of‐care treatment options in this patient population, with > 100 varying regimens identified. Furthermore, patients were often retreated with the same therapies or drug classes from prior LOTs, and patients with TCE and BCMA exposure experienced poor clinical outcomes and rapid treatment discontinuation, as shown by a median TTNT and TTD of 6.8 and 3.5 months, respectively. Collectively, these data emphasise the crucial unmet need for novel, efficacious therapeutics for heavily pretreated patients who have been previously exposed to multiple MM drug classes.

Most patients with MM will experience disease relapse and progression, requiring subsequent therapy [2]. Several retrospective studies have evaluated the real‐world outcomes of heavily pretreated patients with TCE MM. In a real‐world analysis of data from the Centers for Medicare & Medicaid Services Chronic Conditions Data Warehouse, no standard‐of‐care was apparent across any of the four cohorts studied, including TCE, triple‐class–refractory, penta‐exposed, or penta‐exposed and triple‐class–refractory, despite the increasing rate of new patients in each cohort [20]. In an expanded analysis of this database, clinical outcomes were poor, with a median TTD ranging from 4.2–6.9 months. Median TTNT in those who received a subsequent LOT was also poor, ranging from 4.1–5.2 months [21]. Similarly, additional retrospective real‐world analyses using claims and EHR databases reported an estimated median TTD and TTNT of 4.2–5.3 months and 1.7–7.8 months, respectively, among patients with TCE MM with ≥ 4 prior LOTs [4, 5, 6]. These findings are corroborated by other real‐world analyses [22] and collectively underscore the need for additional therapy options for patients with heavily pretreated MM.

Very little is known, however, about the challenges faced by patients with TCE MM who have received subsequent novel therapies, such as BCMA‐targeted therapy, despite this becoming an increasingly prevalent patient population. In one retrospective, multicentre, observational study of heavily pretreated patients with relapsed/refractory MM (RRMM) who had or had not received BCMA‐targeted therapy prior to receiving subsequent ide‐cel therapy, outcomes were suboptimal [23]. Patients who had received prior BCMA therapy were more likely to have penta‐refractory MM and had a higher number of prior LOTs compared to the cohort who had not received prior BCMA therapy before ide‐cel. In this analysis, median progression‐free survival (PFS) was lower for patients with prior BCMA therapy compared to those without (3.2 vs. 9.0 months, respectively) [23]. Moreover, prior treatment with BCMA therapy before ide‐cel was found to be an independent predictor for inferior PFS and OS [23]. In addition, a registry‐based, multicentre, observational study of heavily pretreated patients with RRMM who underwent ide‐cel as part of an early access programme in France showed a similar trend; according to a multivariate analysis of PFS, prior use of BCMA therapy was associated with significantly shorter median PFS (∼3 months) compared with the overall cohort after ide‐cel (12.5 months) [24]. The results of these studies corroborate those reported here in patients with TCE‐ and BCMA‐exposed heavily pretreated MM, as made evident by a median TTNT and TTD of 6.8 and 3.5 months, respectively, thus highlighting the poor clinical outcomes of this patient population.

Another key finding of this real‐world analysis is the substantial heterogeneity in subsequent therapies and lack of any clear standard‐of‐care treatment. This is consistent with observations of prior studies. For instance, in the LocoMMotion prospective real‐world study, 134 unique regimens were identified as subsequent LOTs used by TCE patients with MM, including a range of doublets and triplets [25]. In the current study, 137 subsequent regimens were identified. Of significance, more than three‐fourths of the unique regimens identified here were used by < 5 patients, thus emphasising the lack of established standard‐of‐care therapy. Furthermore, most patients were retreated with the same drugs or drug classes despite prior exposure. Considering this finding, there is a crucial, unmet need for novel treatments in patients with TCE and BCMA exposure.

A notable strength of the current study is that it is the first, large‐scale, real‐world study providing valuable insight into treatment patterns and outcomes among patients with TCE‐ and BCMA‐exposed MM who started a subsequent LOT—a unique, yet growing, patient population that has been scarcely investigated. Furthermore, this study utilised Komodo's Healthcare Map, including a large sample of contemporary real‐world MM patients with various insurance coverages across geographically diverse regions and all 50 states, thus making results more generalisable to patients across the United States.

There are some inherent limitations of this study. For instance, administrative claims data may have contained coding errors or omissions, as they are primarily intended for billing purposes and not necessarily for research. In addition, prescription claim dates reflect when a medication was filled, not necessarily when treatment began. Furthermore, OS data were potentially overestimated in this study due to the incomplete capture of death data; for example, from 2018–2022, Komodo's Healthcare Map captured an average of 78% of deaths compared to the numbers reported by the Centers for Disease Control and Prevention [26]. This is a significant under‐capture of deaths, leading to unrealistic OS estimates. Another limitation of this analysis is the time period during which it was conducted; some of the newer therapies (e.g., anti‐GPRC5D therapies) were not widely available compared to common drug classes at that time. As newer therapies are now available for heavily pretreated patients and given the withdrawal of the US indication of belantamab for patients with TCE RRMM and ≥ 4 prior LOTs [10], it is possible that these findings may evolve in the future.

Open claims were included in this analysis. Due to lack of enrolment data from open claims sources, a limitation is the potential for incomplete capture of claims during a patient's treatment period. Thus, we performed a sensitivity analysis where continuous enrolment was required. There were some differences in baseline characteristics between the main cohort and the smaller continuous enrolment cohort (e.g., region and payment type), which reflects differences in patients represented by Komodo Health's open and closed claims sources. However, TTNT and TTD were similar. The criteria requiring TCE plus BCMA exposure and ≥ 4 prior LOTs likely resulted in selecting patients with sufficient claims activity, thus allowing this study to include patients with data from open claims while mitigating the limitation of open claims on key clinical outcomes. Future studies of late‐line patients with MM may consider similar activity requirements to enable large‐scale, real‐world studies in claims or EHR data and mitigate the risk of missing data in the absence of enrolment data. In addition, a comparative analysis of patients who, in the index LOT, were retreated with drugs or drug classes they were previously exposed to versus those who received drugs or drug classes they were not previously exposed to could be of interest to better understand patient treatment patterns and outcomes. Furthermore, with a sufficient sample size, an analysis comparing patients who were previously treated with different types of BCMA‐targeted therapies (antibody‐drug conjugates vs. T‐cell‐redirecting therapies [i.e., CAR‐T therapies and bispecific antibodies]) would provide valuable insight into treatment patterns and patient outcomes based on different mechanisms of action.

In conclusion, despite advancements in MM therapies and treatment strategies, patients with heavily pretreated TCE‐ and BCMA‐exposed MM continue to experience poor real‐world clinical outcomes. This is demonstrated by rapid treatment discontinuation and the prompt transition to subsequent treatments shortly after initiating subsequent therapy. Moreover, there is no clearly established standard‐of‐care for these patients. These data underscore the unmet need for novel, efficacious therapeutics for patients who have been previously exposed to multiple classes of MM drugs and emphasise the importance of identifying optimal treatment strategies that promote positive long‐term outcomes.

## Author Contributions

H.S.M., J.S.H., H.H.L., A.Z.F., X.Z. and R.F. contributed to the study concept and design. J.S.H. had full access to the data in the study, conducted the study, and takes responsibility for the accuracy of the analysis. All authors contributed to the interpretation of results, participated in drafting and revising the manuscript, and approved the final version for submission.

## Ethics Statement

This study was exempt from review by an institutional review board as the database used was compliant with the Health Insurance Portability and Accountability Act (HIPAA), the study did not involve interaction or interview with any patients and did not include any individually identifiable data.

## Consent

This research was defined as not involving human subjects, as defined in 45 CFR 46.102(f)(2). Therefore, since this study used existing, fully deidentified data and the patients cannot be identified, directly or through identifiers linked to patients, this study was exempt from all 45 CFR part 46 requirements, such as the requirements of informed consent.

## Conflicts of Interest

H.S.M. received honoraria for consultancy from Amgen, Bristol Myers Squibb, FORUS Therapeutics Inc., Janssen, Pfizer, Sanofi and Takeda; and received research funding from Janssen and Pfizer. J.S.H. is a current employee and equity holder of Johnson & Johnson and holds a patent assigned to Johnson & Johnson. H.H.L. was an employee of Johnson & Johnson at the time of the study and held stock options. H.H.L. is a current employee of Takeda. A.Z.F. is a current employee of Johnson & Johnson and holds stock options. S.P. was an employee of Johnson & Johnson at the time of the study and held stock options. S.P. is a current employee of AbbVie. X.Z. is a current employee of Johnson & Johnson and holds stock options. R.F. served as a consultant for AbbVie, Adaptive Biotechnologies, Amgen, Apple, Bristol Meyers Squibb/Celgene, GSK, Janssen, Karyopharm, Pfizer, RA Capital, Regeneron and Sanofi; served on a scientific advisory board for Caris Life Sciences; served on the board of directors for Antengene; and has a current patent for fluorescence in situ hybridisation in multiple myeloma (∼$2000/year).

## Supporting information




**Table S1**:  Index LOT retreatment patterns^a,b,c^ in patients with MM with prior TCE+BCMA exposure and ≥ 4 prior LOTs. **Table S2**:  Treatment history in patients with MM with prior TCE+BCMA exposure and ≥ 4 prior LOTs who received BCMA therapy in the index LOT. **Table S3**:  Cohort characteristics and outcomes with and without continuous enrolment.^a^ **Figure S1**:  Summary of attrition and first BCMA‐targeted therapy for patients with MM with prior TCE+BCMA exposure and ≥ 4 prior LOTs (Attrition Steps 2–3 in Figure 2).^a^ **Figure S2**:  TTNT and TTD in patients with MM with prior TCE+BCMA exposure and ≥ 4 prior LOTs who received BCMA therapy in the index LOT. **Figure S3**:  TTNT and TTD in patients with MM with prior TCE+BCMA exposure and ≥ 4 prior LOTs who were not penta‐exposed. **Figure S4**:  TTNT and TTD in patients with MM with prior TCE+BCMA exposure and ≥ 4 prior LOTs who were penta‐exposed.

## Data Availability

These data were made available by Komodo Health and used under licence for the current study and are not publicly available. The data are accessible to other researchers by contacting Komodo Health at https://www.komodohealth.com/contact‐us.
