# Supplementary material for: Real‐World Treatment Patterns and Clinical Outcomes Among Patients With Triple‐Class–Exposed and BCMA‐Exposed Multiple Myeloma Within the United States
Source: EJHaem. 2025 Sep 23;6(5):e70145. doi: 10.1002/jha2.70145 (PMC12455680; doi:10.1002/jha2.70145)
Supplement: Supplementary file 1 — Table S1: Index LOT retreatment patternsa,b,c in patients with MM with prior TCE+BCMA exposure and ≥ 4 prior LOTs. Table S2: Treatment history in patients with MM with prior TCE+BCMA exposure and ≥ 4 prior LOTs who received BCMA therapy in the index LOT. Table S3: Cohort characteristics and outcomes with and without continuous enrolment.a Figure S1: Summary of attrition and first BCMA‐targeted therapy for patients with MM with prior TCE+BCMA exposure and ≥ 4 prior LOTs (Attrition Steps 2–3 in Figure 2).a Figure S2: TTNT and TTD in patients with MM with prior TCE+BCMA exposure and ≥ 4 prior LOTs who received BCMA therapy in the index LOT. Figure S3: TTNT and TTD in patients with MM with prior TCE+BCMA exposure and ≥ 4 prior LOTs who were not penta‐exposed. Figure S4: TTNT and TTD in patients with MM with prior TCE+BCMA exposure and ≥ 4 prior LOTs who were penta‐exposed. [file JHA2-6-e70145-s001.docx]

# Supplemental Appendix

Real-world Treatment Patterns and Clinical Outcomes Among Patients with Triple-class–exposed and BCMA-exposed Multiple Myeloma Within the US

# Table S1. Index LOT retreatment patterns^a,b,c^ in patients with MM with prior TCE + BCMA exposure and ≥4 prior LOTs

|  | **Patients retreated^b^**  **(n/n [%])** |
| --- | --- |
| Most common drug classes within index regimen,^d^ % |  |
| PI | 243/243 (100.0) |
| BCMA | 186/186 (100.0) |
| IMiD | 155/155 (100.0) |
| Anti-CD38 mAb | 102/102 (100.0) |
| Chemotherapy | 80/134 (59.7) |
| SINE | <11/103 (<10.7) |
| Anti-CS1 | <11/39 (<28.2) |

LOT, line of therapy; MM, multiple myeloma; TCE, triple-class–exposed; BCMA, B-cell maturation antigen; PI, proteasome inhibitor; IMiD, immunomodulatory drug; SINE, selective inhibitor of nuclear export; mAb, monoclonal antibody.

^a^Among patients with ≥3 months post-index activity (unless death was observed before last claim date; n=633).

^b^Patients with a drug class within the main index LOT regimen (denominator) who were previously treated with the same drug class at any time before index (numerator).

^c^Excluding corticosteroids.

^d^Most common defined as a frequency of ≥5% of patients, as presented in Table 2 (n=633).

# Table S2. Treatment history in patients with MM with prior TCE + BCMA exposure and ≥4 prior LOTs who received BCMA therapy in the index LOT

|  | **BCMA in index LOT**  **(n=191)** |
| --- | --- |
| Mean (SD) age, years | 65.4 (9.8) |
| Sex, n (%) |  |
| Male | 108 (56.5) |
| Female | 83 (43.5) |
| Race, n (%) |  |
| White | 113 (59.2) |
| Black | 36 (18.8) |
| Hispanic or Latino | 14 (7.3) |
| Asian or Pacific Islander | <11 (<5.8)^a^ |
| Other or unknown | <11 (<5.8)^a^ |
| US region, n (%) |  |
| Northeast | 60 (31.4) |
| South | 49 (25.7) |
| Midwest | 46 (24.1) |
| West | 26 (13.6) |
| Other^b^ | <11 (<5.8)^a^ |
| Mean (SD) QCCI score | 3.9 (3.3) |
| Comorbidities, n (%) |  |
| Hypertension | 117 (61.3) |
| Any CRAB symptom | 115 (60.2) |
| Peripheral neuropathy | 77 (40.3) |
| Cardiovascular conditions | 62 (32.5) |
| Diabetes | 54 (28.3) |
| Mean (SD) time from first MM diagnosis to index date, years | 5.6 (1.7) |
| Mean (SD) prior LOTs before index date | 6.0 (1.6) |
| Mean (SD) time from prior LOT end date to index date, months | 5.1 (4.7) |
| Prior treatments before index date |  |
| Prior PI, n (%) |  |
| Carfilzomib | 172 (90.1) |
| Bortezomib | 152 (79.6) |
| Ixazomib | 54 (28.3) |
| Prior IMiD, n (%) |  |
| Pomalidomide | 169 (88.5) |
| Lenalidomide | 138 (72.3) |
| Thalidomide | 15 (7.9) |
| Prior anti-CD38 mAb, n (%) |  |
| Daratumumab | 186 (97.4) |
| Isatuximab | 25 (13.1) |
| Prior BCMA, n (%) |  |
| Belantamab | 140 (73.3) |
| Mean (SD) duration of prior belantamab treatment, days | 114 (142.6) |
| Ide-cel | 38 (19.9) |
| Unspecified CAR-T^c^ | <11 (<5.8)^a^ |
| Teclistamab | <11 (<5.8)^a^ |
| Cilta-cel | <11 (<5.8)^a^ |
| Prior selinexor, n (%) | 52 (27.2) |
| Prior SCT, n (%) | 110 (57.6) |
| Penta-drug exposed,^d^ n (%) | 101 (52.9) |

TCE, triple-class–exposed; BCMA, B-cell maturation antigen; LOT, line of therapy; SD, standard deviation; US, United States; QCCI, Quan-Charlson Comorbidity Index; CRAB, hypercalcemia, renal failure, anemia, bone lesions; MM, multiple myeloma; PI, proteasome inhibitor; IMiD, immunomodulatory drug; mAb, monoclonal antibody; belantamab, belantamab mafodotin; ide-cel, idecabtagene vicleucel; cilta-cel, ciltacabtagene autoleucel; SCT, stem cell transplant.

^a^Due to Komodo Health’s patient de-identification policies, data including 1-10 patients were masked.

^b^Other is defined as 2+ regions.

^c^Ide-cel or cilta-cel.

^d^Defined as having received ≥2 PIs, ≥2 IMiDs, and ≥1 anti-CD38 therapy.

# Table S3. Cohort characteristics and outcomes with and without continuous enrolment.^a^

|  | **TCE + BCMA** | |
| --- | --- | --- |
|  | **With CE** | **Without CE** |
| Patients | n=194 | n=656 |
| Mean age, years | 64.6 | 66.5 |
| Age category, % |  |  |
| <65 | 51.5 | 39.0 |
| 65-69 | 17.0 | 20.7 |
| 70-74 | 11.9 | 17.5 |
| ≥75 | 19.6 | 22.7 |
| Sex, % |  |  |
| Female | 40.7 | 42.1 |
| Race, % |  |  |
| White | 57.2 | 60.5 |
| Region, % |  |  |
| West | 10.8 | 13.0 |
| Northeast | 28.9 | 25.2 |
| Midwest | 28.9 | 21.2 |
| South | 24.7 | 35.4 |
| Mean QCCI score | 4.3 | 3.7 |
| Prior therapy |  |  |
| Mean prior LOTs before index | 5.4 | 5.9 |
| Prior carfilzomib, % | 92.3 | 85.8 |
| Prior pomalidomide, % | 91.8 | 86.0 |
| Prior daratumumab, % | 98.5 | 98.5 |
| Prior SCT, % | 61.3 | 50.8 |
| Time-to-event outcomes |  |  |
| Median TTD, months | 3.4 | 3.5 |
| Median TTNT, months | 6.9 | 6.8 |
| Median follow-up time,^b^ months | 7.9 | 16.0 |

TCE, triple-class–exposed; BCMA, B-cell maturation antigen; CE, continuous enrolment; QCCI, Quan-Charlson Comorbidity Index; LOT, line of therapy; SCT, stem cell transplant; TTD, time to discontinuation; TTNT, time to next treatment.

^a^Continuous enrolment was defined as enrolment in both medical and pharmacy plans from 6 months before the LOT start date through the LOT start date (baseline period).

^b^In cohorts without CE, follow-up ended on the earliest of study end date, last claim date, or death date; in cohorts with CE, follow-up may have ended earlier on CE end date or last closed claim date.

**Figure S1. Summary of attrition and first BCMA-targeted therapy for patients with MM with prior TCE + BCMA exposure and ≥4 prior LOTs (Attrition Steps 2 to 3 in Figure 2).^a^**


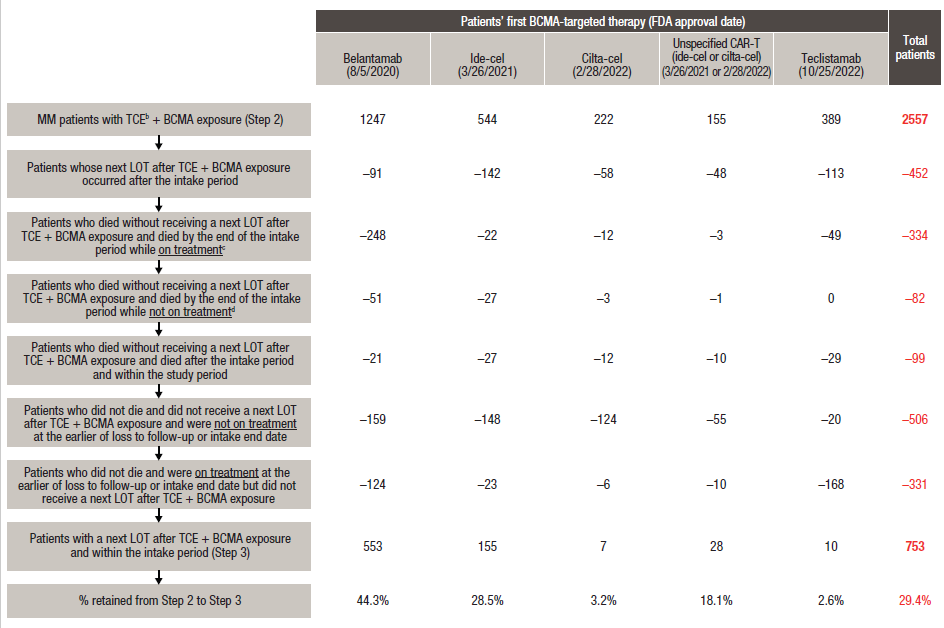


BCMA, B-cell maturation antigen; MM, multiple myeloma; TCE, triple-class–exposed; LOT, line of therapy; FDA, Food and Drug Administration; belantamab, belantamab mafodotin; ide-cel, idecabtagene vicleucel; cilta-cel, ciltacabtagene autoleucel; PI, proteasome inhibitor; IMiD, immunomodulatory drug; mAb, monoclonal antibody.

^a^Retention percentages are calculated from the total number of patients from the previous step.

^b^TCE was defined as ≥1 PI, ≥1 IMiD, and ≥1 anti-CD38 mAb between the study start date and intake end date.

^c^On treatment: follow-up end date was ≤90 days after the last observed date of treatment during which the patient became TCE + BCMA exposed.

^d^Not on treatment: follow-up end date was >90 days after the last observed date of treatment during which the patient became TCE + BCMA exposed.

**Figure S2. TTNT and TTD in patients with MM with prior TCE + BCMA exposure and ≥4 prior LOTs who received BCMA therapy in the index LOT.**


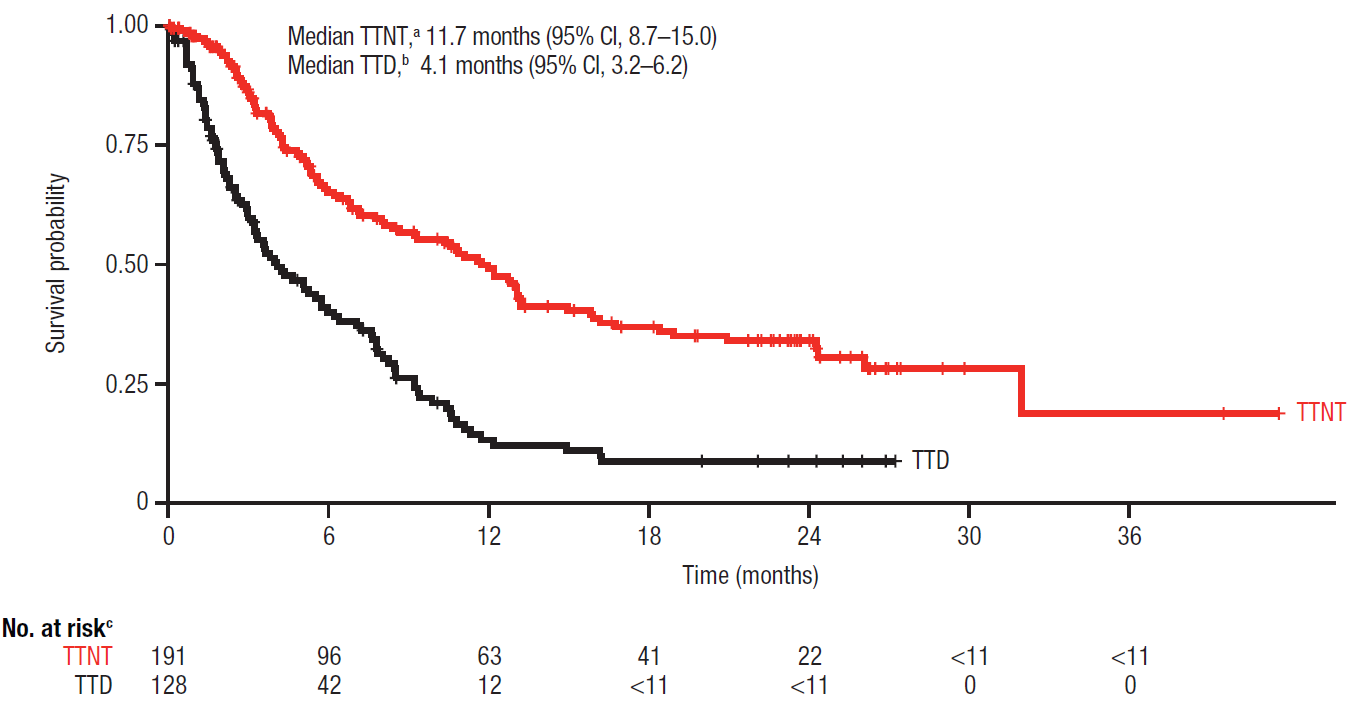


TTNT, time to next treatment; TTD, time to discontinuation; MM, multiple myeloma; TCE, triple-class–exposed; BCMA, B-cell maturation antigen; LOT, line of therapy; CI, confidence interval.

^a^TTNT was defined as the time from the index date to death or the initiation of a next LOT.

^b^TTD was defined as the time from the index date to death or the discontinuation of the index LOT.

^c^Due to Komodo Health’s patient de-identification policies, data including 1-10 patients were masked.

**Figure S3. TTNT and TTD in patients with MM with prior TCE + BCMA exposure and ≥4 prior LOTs who were not penta-exposed.**


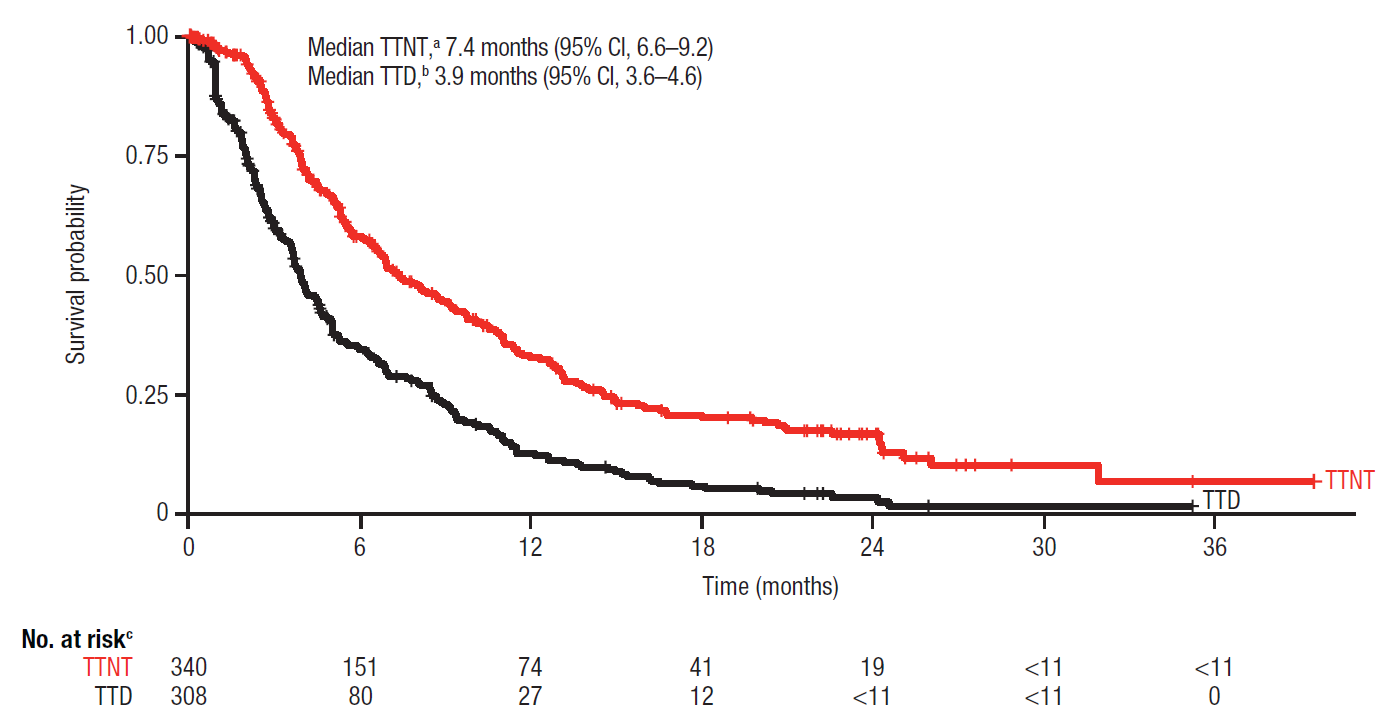


TTNT, time to next treatment; TTD, time to discontinuation; MM, multiple myeloma; TCE, triple-class–exposed; BCMA, B-cell maturation antigen; LOT, line of therapy; CI, confidence interval.

^a^TTNT was defined as the time from the index date to death or the initiation of a next LOT.

^b^TTD was defined as the time from the index date to death or the discontinuation of the index LOT.

^c^Due to Komodo Health’s patient de-identification policies, data including 1-10 patients were masked.

**Figure S4. TTNT and TTD in patients with MM with prior TCE + BCMA exposure and ≥4 prior LOTs who were penta-exposed.**


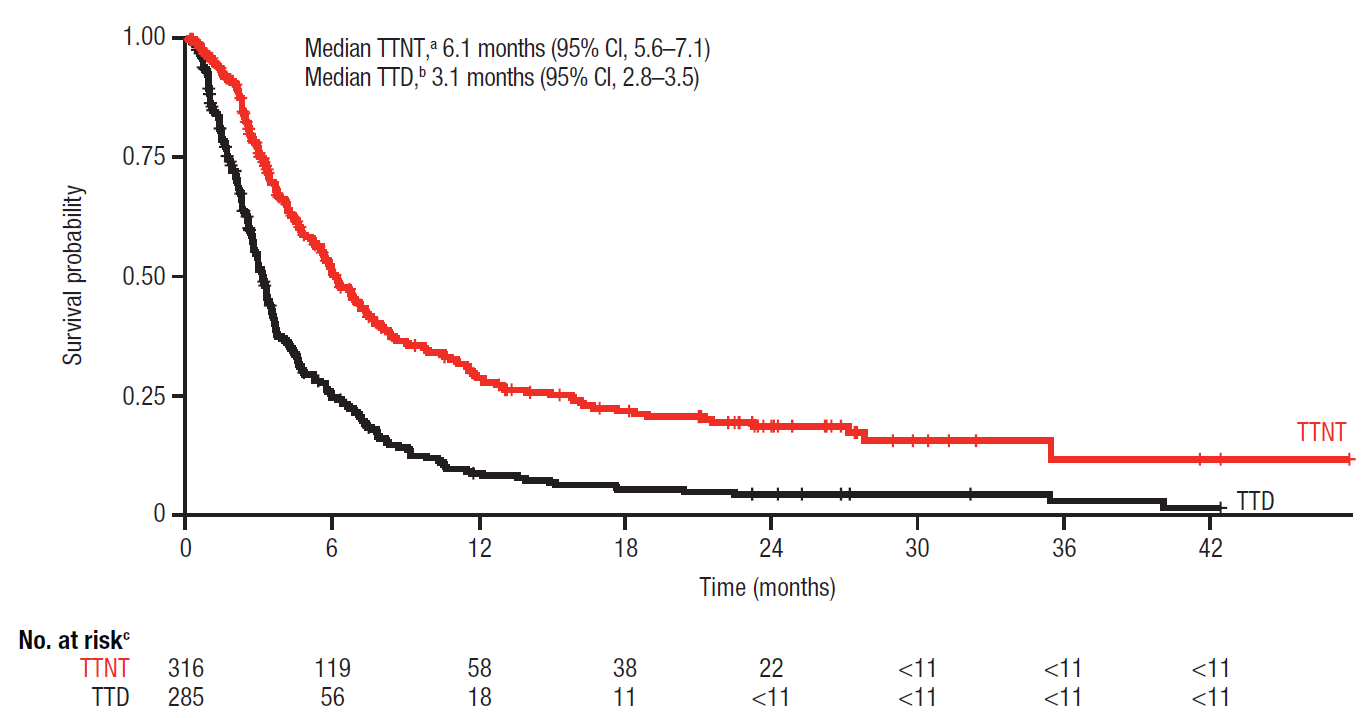


TTNT, time to next treatment; TTD, time to discontinuation; MM, multiple myeloma; TCE, triple-class–exposed; BCMA, B-cell maturation antigen; LOT, line of therapy; CI, confidence interval.

^a^TTNT was defined as the time from the index date to death or the initiation of a next LOT.

^b^TTD was defined as the time from the index date to death or the discontinuation of the index LOT.

^c^Due to Komodo Health’s patient de-identification policies, data including 1-10 patients were masked.
